# Iron and Chronic Kidney Disease: Still a Challenge

**DOI:** 10.3389/fmed.2020.565135

**Published:** 2020-12-18

**Authors:** Ewa Wojtaszek, Tomasz Glogowski, Jolanta Malyszko

**Affiliations:** Department of Nephrology, Dialysis and Internal Diseases, The Medical University of Warsaw, Warsaw, Poland

**Keywords:** iron, chronic kidney disease, hepcidin, erythroferrone, HIF-1α, anemia

## Abstract

Anemia is a clinical feature of chronic kidney disease (CKD). Most common causes are iron and erythropoietin deficiency. The last two decades have yielded significant advances in understanding iron balance's physiology, including iron trafficking and the crosstalk between iron, oxygen, and erythropoiesis. This knowledge sheds new light on the regulation and disturbance of iron homeostasis in CKD and holds the promise for developing new diagnostic and therapeutic tools to improve the management of iron disorders. Hepcidin–ferroportin axis has a central role in regulating body iron balance and coordinating communication between tissues and cells that acquire, store, and utilize iron. Recent research has revealed a bidirectional relationship between fibroblast growth factor 23 (FGF23) and iron status, anemia, and inflammation, as well as the role of erythroferrone (ERFE) in iron homeostasis. However, ERFE concentrations and actions are not well-characterized in CKD patients. Studies on ERFE in CKD are limited with slightly conflicting results. Despite general interest in iron metabolism in kidney diseases, studies on the less prevalent renal replacement therapy mode, such as peritoneal dialysis or hemodiafiltration, are scarce. Slightly more was published on hemodialysis. There are several novel options on the horizon; however, clinical data are limited. One should be aware of the potential risks and benefits of the novel, sophisticated therapies. An inhibition of hepcidin on the different pathways might be also a viable adjunctive therapeutic option in other clinical situations.

## Introduction

Anemia is an important complication of chronic kidney disease (CKD), with increasing prevalence in the more advanced stages of the disease (1). The etiology of anemia in CKD is multifactorial, and the key mechanisms involve relative deficiency of erythropoietin (EPO), iron deficiency and maldistribution, and shortened erythrocyte life span (2, 3).

The prevalence of iron deficiency anemia (IDA) increases along with the progression of kidney disease and affects ~3 quarters of patients with estimated glomerular filtration rate (eGFR) <15 ml/min/1.73 m^2^ (1, 3, 4). IDA in CKD patients often results from absolute and functional iron deficiency and is associated with adverse outcomes in these patients (5). Moreover, it has been shown that iron deficiency, as such, is highly prevalent in CKD patients and is associated with an increased risk of morbidity and mortality, independent of potential confounders, including anemia (6–9). Therefore, the repletion of iron stores seems mandatory in the treatment of IDA, and it maximizes the efficacy of EPO-stimulating agents (ESAs), considered a staple in the management of anemia in CKD patients. However, iron homeostasis's traditional biomarkers used to detect iron deficiency in CKD patients are often imprecise and unreliable, rendering the diagnostic, therapeutic, and monitoring processes difficult. In this context, to best manage IDA in CKD, a thorough understanding of iron homeostasis's physiology and pathophysiology and its disturbances is essential.

The last two decades have yielded significant advances in understanding the physiology of iron balance, including iron trafficking and the crosstalk between iron, oxygen, and erythropoiesis. This knowledge sheds new light on the regulation and disturbance of iron homeostasis in CKD and holds the promise for developing new diagnostic and therapeutic tools to improve the management of iron disorders (10, 11).

This review summarizes recent advances in understanding iron homeostasis and the crosstalk between iron and erythropoiesis in CKD, and the possible therapeutic options in renal anemia.

## Physiology of Iron Metabolism

Iron and erythropoiesis are connected at multiple levels and reciprocally regulated. Iron tunes renal synthesis of EPO mediated by hypoxia-inducible factor-2α (HIF-2α). EPO-stimulated erythroblasts release erythroferrone (ERFE), which suppress hepcidin expression and promotes the acquisition of iron necessary for smooth maturation of erythroblasts to erythrocytes (12, 13).

### Erythropoiesis

**Figure 1:**
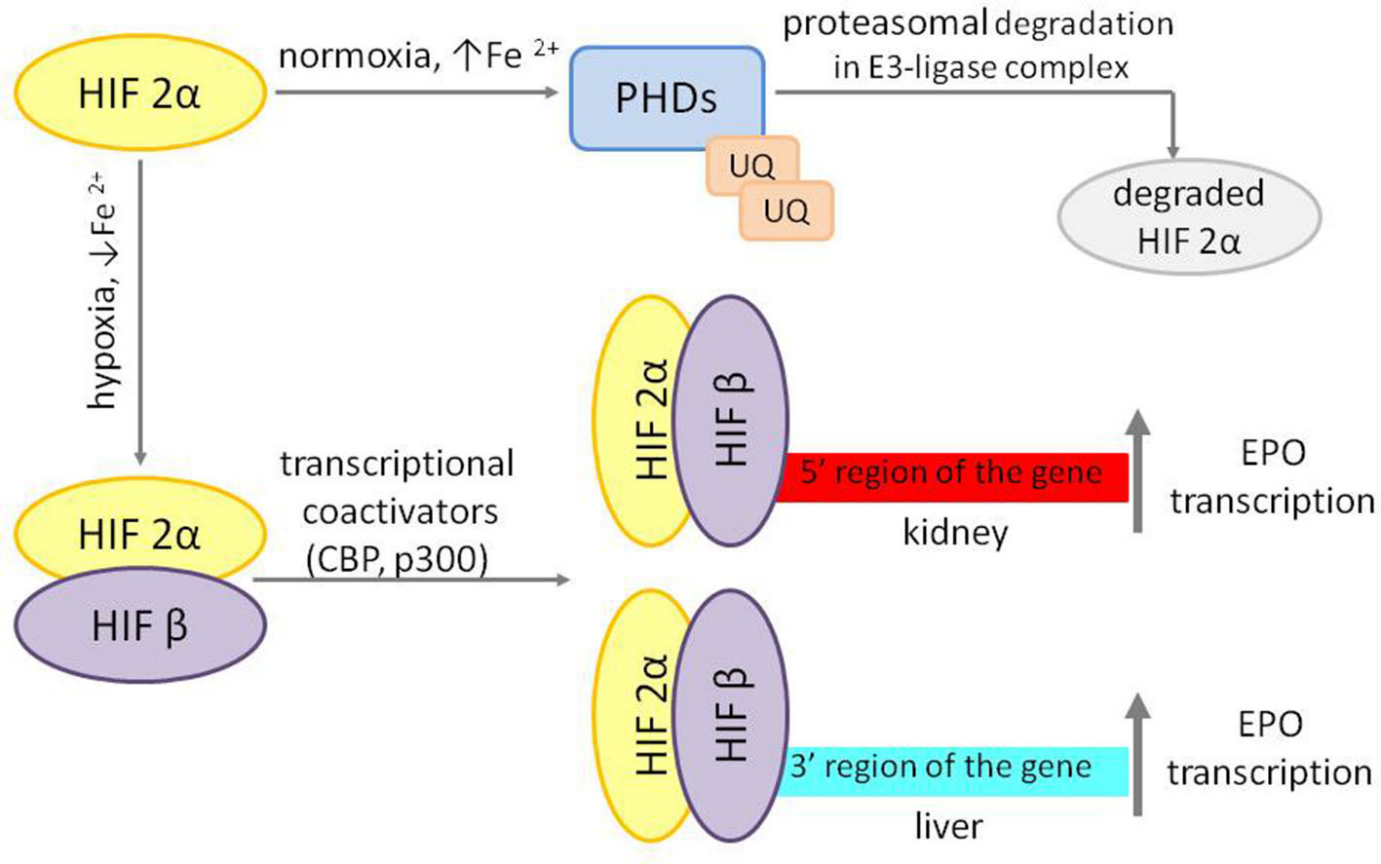
Regulation of EPO transcription. HIF 2α, hypoxia-inducible factor 2α; HIF β, hypoxia-inducible factor β; PDHs, O_2_ and iron-dependent HIF prolyl-4-hydroxylases; UQ, ubiquitin; CBP, CREB-binding protein; EPO, erythropoietin.

In adults, EPO is produced in the kidneys by endothelial interstitial fibroblasts in the renal cortex and the outer medulla (14) in response to the tissues' oxygen pressure and regulated by the transcription factor HIF-2α. Under sufficient oxygen supply, iron oxygen-dependent prolyl-hydroxylases (HIF-PHDs) cause hydroxylation of HIF-2α, which is subsequently degraded in proteasomes. In a hypoxia state, regardless of its cause and iron deficiency, HIF-2α is stabilized and translocated to the nucleus, where it forms heterodimers with HIF-β or aryl hydrocarbon receptor nucleus translocator (ARNT). HIF-2α/β heterodimers with coactivators bind to consensus elements of the gene (5′ for the kidney) or (3′ for the liver) and initiate EPO transcription (15) (Figure 1).

### Erythroferrone—The Link Between Erythropoiesis and Iron Metabolism

**Figure 2:**
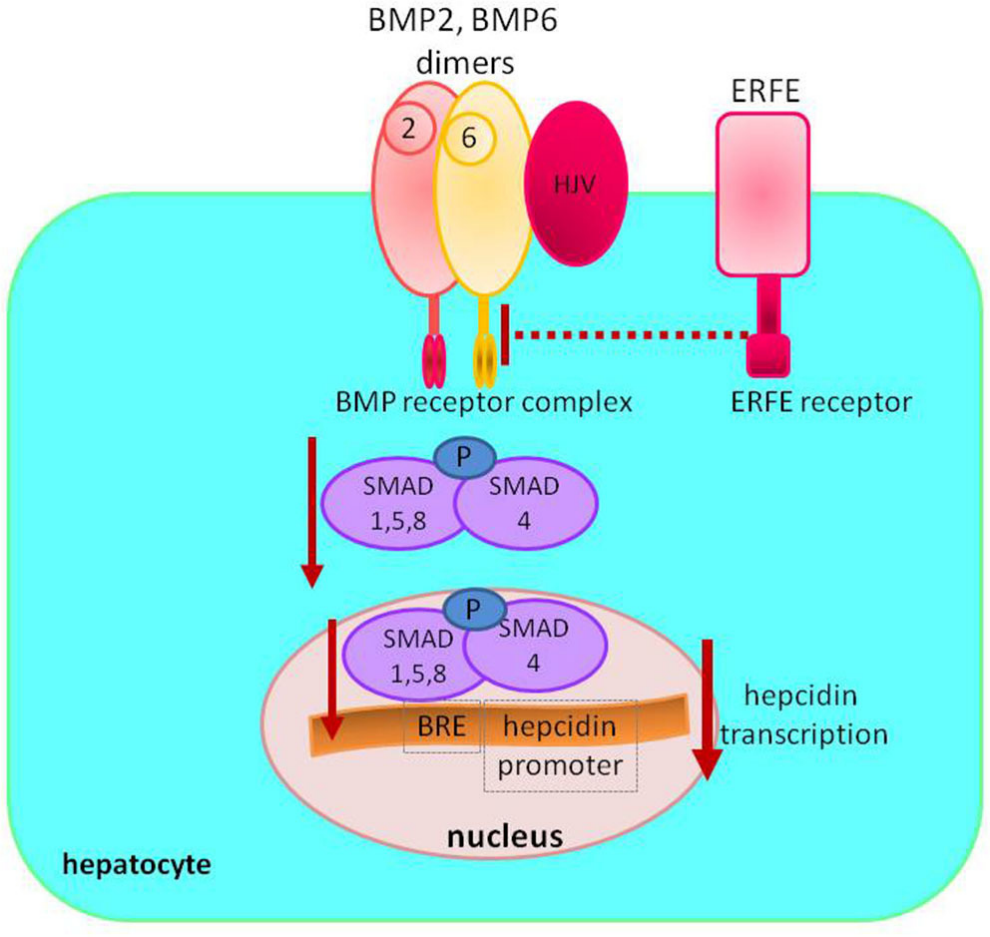
Regulation of hepcidin synthesis by erythroferrone. Increased circulating levels of ERFE sequestrate BMP receptor ligands to decrease SMAD1/5/8 phosphorylation; lower levels of phosphorylated SMAD1/5/8 result in less SMAD1/5/8–SMAD4 complex formation, diminishing its nuclear concentration and decreasing hepcidin transcription. ERFE, erythroferrone; BMP, bone morphogenetic protein; SMAD, suppressor of mothers against decapentaplegic proteins; HJV, hemojuvelin; BRE, BMP-responsive elements in the hepcidin promoter; P, phosphate.

Increased erythropoietic activity in response to anemia or exogenous EPO administration enhances ERFE synthesis by erythroblasts. ERFE inhibits hepcidin production, which increases iron absorption and its mobilization from iron stores (Figure 2).

Hepcidin suppression in response to EPO is mediated through suppressor of mothers against decapentaplegic (SMAD) proteins signaling. ERFE sequestrates bone morphogenetic protein (BMP) receptor ligands, especially BMP6, and suppress SMAD1/5 phosphorylation in Hep3b cells (16, 17).

### Hepcidin–Ferroportin Axis—A Key Regulator of Iron Balance

Hepcidin–ferroportin axis has a central role in regulating body iron balance and coordinating acquirement, storage, and utilization of iron at the systemic level.

**Figure 3:**
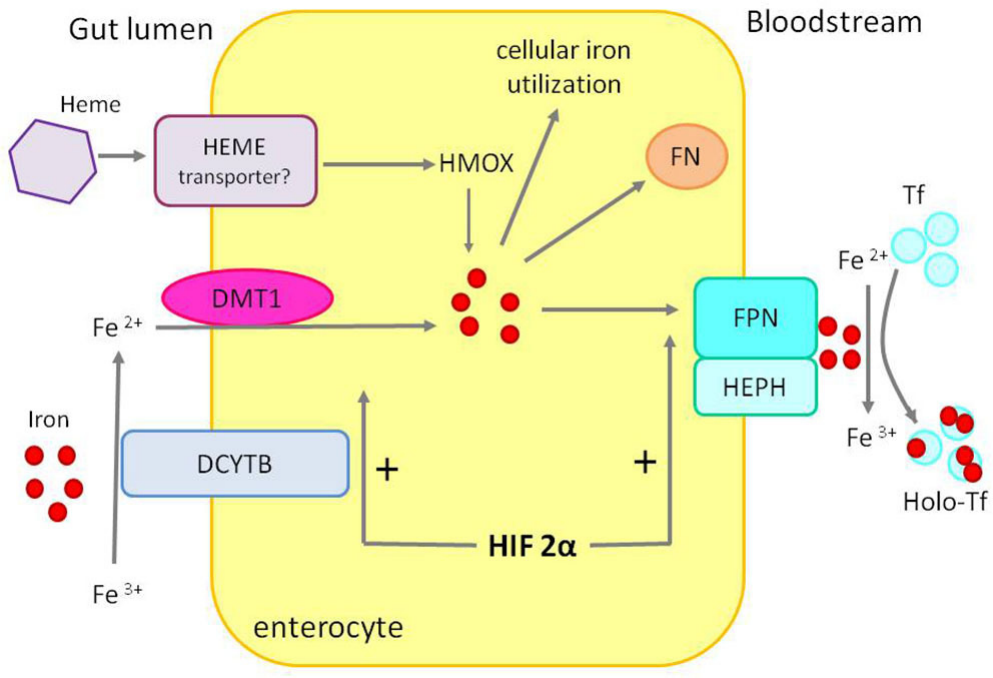
Duodenal iron absorption. The iron transporter DMT1 takes up ferrous iron, reduced from ferric iron by DCYTB, on the enterocyte's luminal. Iron can be used inside the cell, exported to circulation by FPN after ferrous iron is oxidized to ferric iron by HEPH or stored in FN. Heme, after entering the enterocyte through an unknown mechanism, is converted to iron by HMOX. HIF 2α stimulates the expression of DMT1 and FPN—apical and basolateral transporters. DCYTB, duodenal cytochrome B reductase; DTM1, divalent metal transporter 1; FPN, ferroportin; HEPH, hephaestin; HMOX, heme oxygenase; FN, ferritin; Tf, transferrin; Holo-Tf, holotransferrin.

Dietary iron absorption occurs primarily in the duodenum. Non-heme iron, after reduction from ferric to ferrous form by duodenal cytochrome B reductase (DCYTB), is imported from the gut lumen by the divalent metal transporter 1 (DMT1) located at the apical surface of the duodenal enterocyte. Absorbed iron may be utilized by the enterocyte, stored in ferritin, or exported into circulation via ferroportin located at the enterocyte's basolateral membrane (Figure 3).

Iron absorption is an example of crosstalk between hypoxia and the hepcidin–ferroportin axis. In hypoxic environment, translation of HIF 2α [controlled by iron regulatory protein 1 (IRP1)] upregulates the expression of apical (DMT1) and basolateral [ferroportin (FPN)] enterocyte iron transporters. In iron deficiency, absorption is enhanced by hepcidin downregulation, stabilizing HIF-2α and favoring iron export, and, on the contrary, iron overload and high hepcidin impair luminal iron uptake (18, 19).

**Figure 4:**
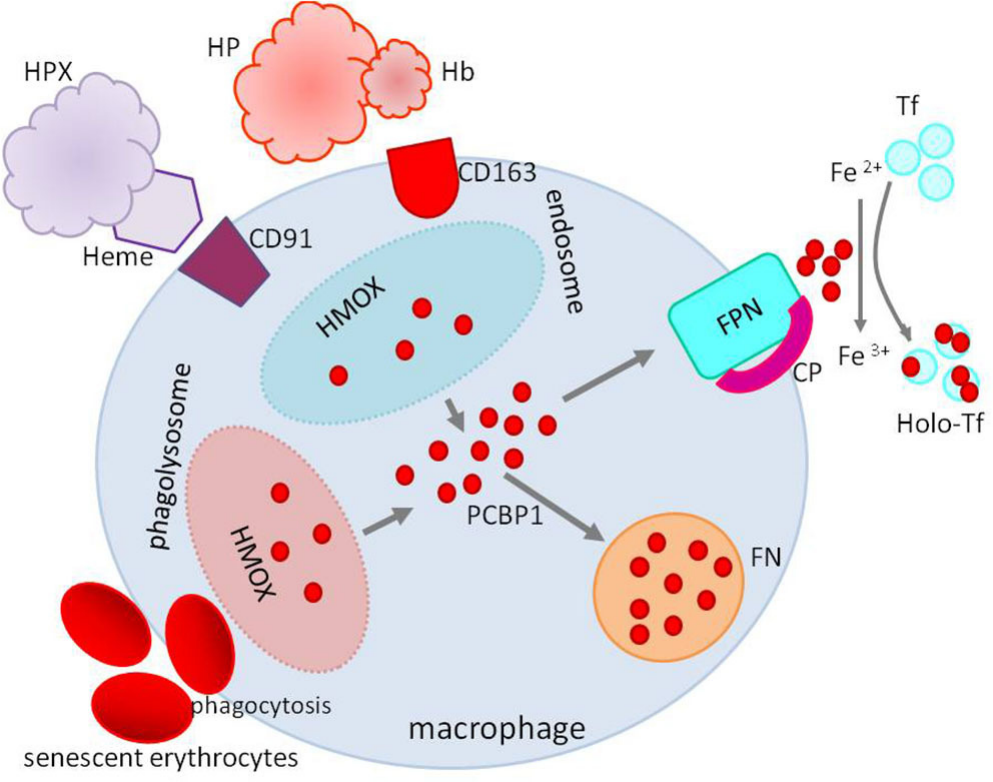
Iron recycling in macrophages. Macrophages recover iron from phagocytized erythrocytes and heme from heme–HPX or HP–hemoglobin complexes after degradation by HMOX. Iron not used in the macrophage is stored in FN or exported to the circulation by FPN with CP's cooperation. Heme–HPX, heme–hemopexin; HP-Hb, haptoglobin–hemoglobin; HMOX, heme oxygenase; FPN, ferroportin; CP, ceruloplasmin; FN, ferritin; Tf, transferrin; Holo-Tf, holotransferrin; PCBP1, poly(rC)-binding protein 1.

However, the primary sources of daily iron are iron recycling macrophages, phagocytizing aging, and damaged erythrocytes and recovering iron from heme by oxygenase 1. Macrophages can use, preserve, and export iron. Macrophage ferroportin responsible for iron export is crucial for iron homeostasis. Its expression is downregulated by inflammatory cytokines contributing to iron sequestration, and its translation is repressed by iron and, at the post-translational level, controlled by hepcidin (12, 13) (Figure 4).

Once absorbed or recycled, iron binds in the plasma to the iron carrier, transferrin (Tf), which is essential for efficient iron transfer to target cells. Transferrin, except the function as an iron cargo, acts as an iron regulator, which seems to depend on the unequal ability of iron binding and different interactions with two receptors (13).

Binding to transferrin receptor 1 (TFR1), transferrin delivers iron to cells through the endosomal cycle, which is crucial for erythroblasts, muscle cells, B- and T-lymphocytes, basal iron uptake in hepatocytes, and epithelial homeostasis in the gut (irrespective of its function in iron transport) (10, 13).

By binding to transferrin receptor 2 (TFR2), whose expression is restricted to hepatocytes and erythroblasts and which has lower binding affinity than TFR1, transferrin acts as a regulator of iron homeostasis. In high plasma iron concentration, transferrin binds to TFR2, upregulates hepcidin in hepatocytes, and reduces EPO responsiveness in erythroid cells, where TFR2 binds EPO receptor. In iron, deficiency occurs opposite regulation (10, 13).

By binding iron, transferrin keeps iron in a neutral redox state. Each transferrin molecule can carry up to two iron atoms, occupying about 20–40% of the iron binding sites on transferrin and corresponds to transferrin saturation (TSAT). If serum iron exceeds the transferrin carrying capacity, as is the case with massive blood transfusions or parenteral administration of iron, non-transferrin-bound iron (NTBI), including highly reactive labile plasma iron (LPI), may circulate (20). LPI may pose the risk of tissue damage, both by oxidation products and by non-regulated infiltration into cells leading to iron overload (21).

Iron not utilized by cells, to avoid its toxicity, is bound by poly(rC)-binding protein 1 (PCBP1) and delivered to ferritin. Ferritin may store up to 4,500 iron atoms and serves as an essential source for future needs. Ferritin turnover occurs through an autophagic process coordinated by nuclear receptor co-activator 4 (NCOA)4 that directs ferritin to lysosomal degradation to recover iron (13). The origin and function of serum ferritin are poorly studied. It is hypothesized that cells may re-uptake the secreted ferritin as an alternative mechanism of iron recycling when the iron is blocked in macrophages during inflammation (13).

PCBP1 links iron and hypoxia pathways, delivering iron to prolyl hydroxylase domain enzyme 2 (PHD2), the enzyme inducing degradation of HIFs (22).

### Hepcidin

Hepcidin, a 25 amino acid, cationic peptide secreted by the liver, is a master regulator of systemic iron homeostasis. Hepcidin expression is regulated by iron and inflammatory cytokines but, as recently shown, also by growth factors [e.g., epidermal growth factor (EGF) or hepatocyte growth factor (HGF)], steroid hormones (estrogen and testosterone), and even metabolic factors (e.g., vitamin D or glucose) (11).

Iron, both tissue and circulating, regulates hepcidin transcription. The signal for hepcidin expression is initiated by BMP ligands using hemojuvelin (HJV) as a co-receptor that binds to BMP6 and BMP2 and activate the hepatocyte BMP–SMAD protein pathway (10, 13).

**Figure 5:**
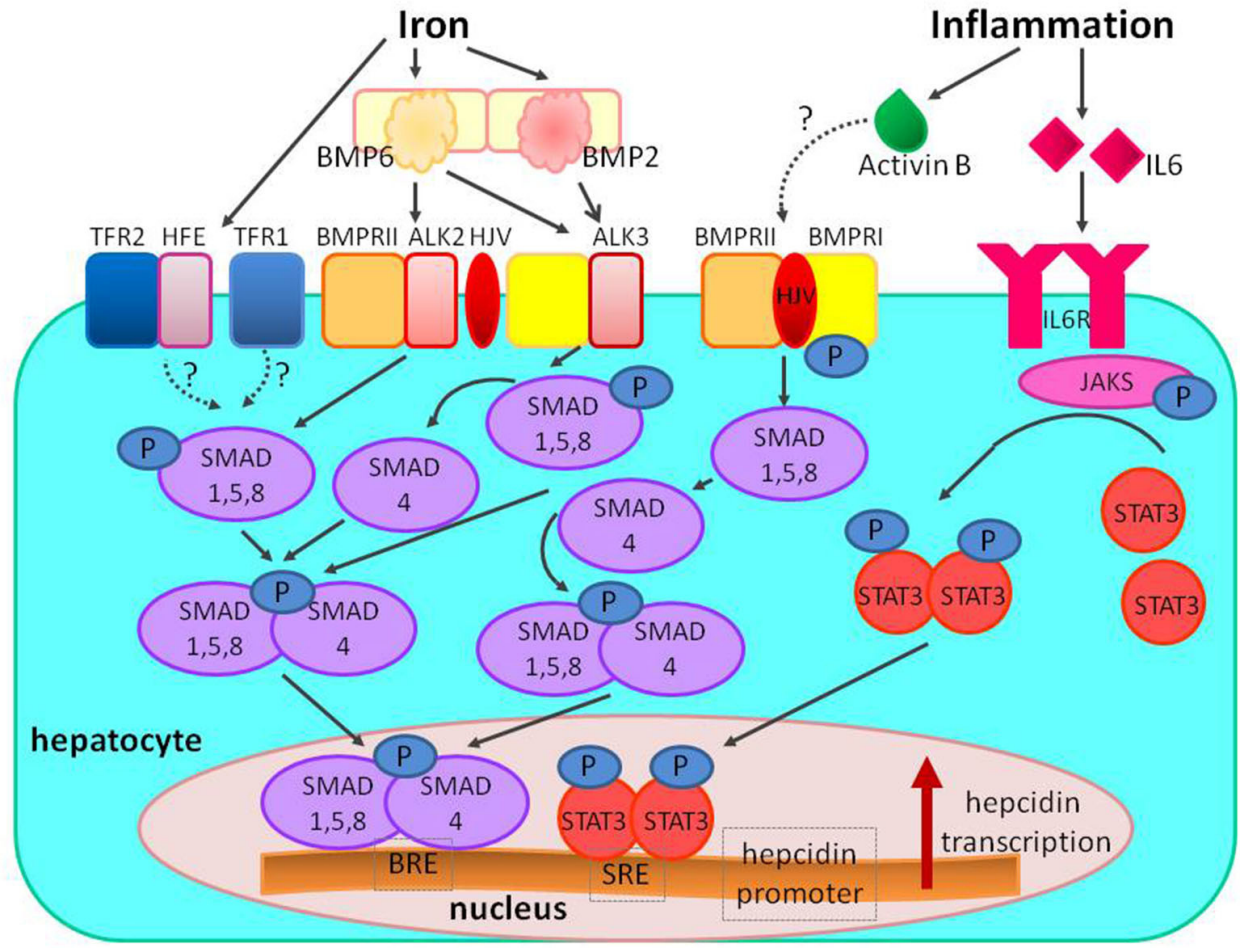
The regulation of hepcidin expression by iron and inflammation. Increased systemic iron stimulates the production of the ligand BMP6, which binds to the BMPRI (ALK2/ALK3) and BMPRII receptors, and the co-receptor HJV to stimulate phosphorylation of the SMAD1/5/8 signaling molecules; phosphorylated SMAD1/5/8 binds to SMAD4 and translocates to the nucleus to bind BRE in the hepcidin promoter. In iron overload diferric transferrin displaces HFE from TFR1 to enable iron uptake and stabilizes TFR2 potentiating SMAD signaling (the role of the other HFE and TFR2 proteins is still unclear). Inflammation also stimulates hepcidin production; inflammation increases IL-6 binding to IL-6R and stimulating phosphorylation of JAKs and STAT3; phosphorylated STAT3 homodimers translocate to nucleus and bind to SRE in hepcidin promoter. The proposed mechanism of interaction between inflammatory and BMP signaling includes activin B, which is induced by inflammation, and binds to BMPRs to stimulate SMAD1/5/8 phosphorylation. The interaction between SMAD and STAT can also take place at the level of hepcidin promoter. BMP, bone morphogenetic protein; BMPR, bone morphogenetic protein receptor; SMAD, suppressor of mothers against decapentaplegic proteins; JAK, Janus kinase; STAT3, signal transducer and activator of transcription; HJV, hemojuvelin; HFE, hemochromatosis protein; IL-6, interleukin 6; IL-6R, interleukin 6 receptor; TfR, transferrin receptor; BRE, BMP-responsive elements in the hepcidin promoter; SRE, STAT-responsive elements in the hepcidin promoter; P, phosphate.

Inflammatory cytokine IL-6 upregulates hepcidin expression by activating IL-6 receptor (IL-6R)–Janus kinase 3 (JAK3)–signal transducer and activator of transcription 3 (STAT3) pathway. For full hepcidin activation, the IL-6 pathway requires functional BMP-SMAD signaling (10, 13), and hypothesized mechanisms for this connection are the interactions between STAT3 and SMADs at the level of hepcidin promoter and activin B (23) (Figure 5).

Hepcidin expression is inhibited by expansion of erythropoiesis (for example, in response to ESA administration) or hypoxia. Hepcidin suppression is mediated via BMP/SMAD signaling pathway by ERFE (16, 17).

## Iron Metabolism in Chronic Kidney Disease

Patients with CKD experience marked alterations in iron balance and distribution, when some cells and tissues are iron deficient and some iron loaded. It leads to dysregulation of physiological crosstalk between iron, oxygen, and erythropoiesis.

**Figure 6:**
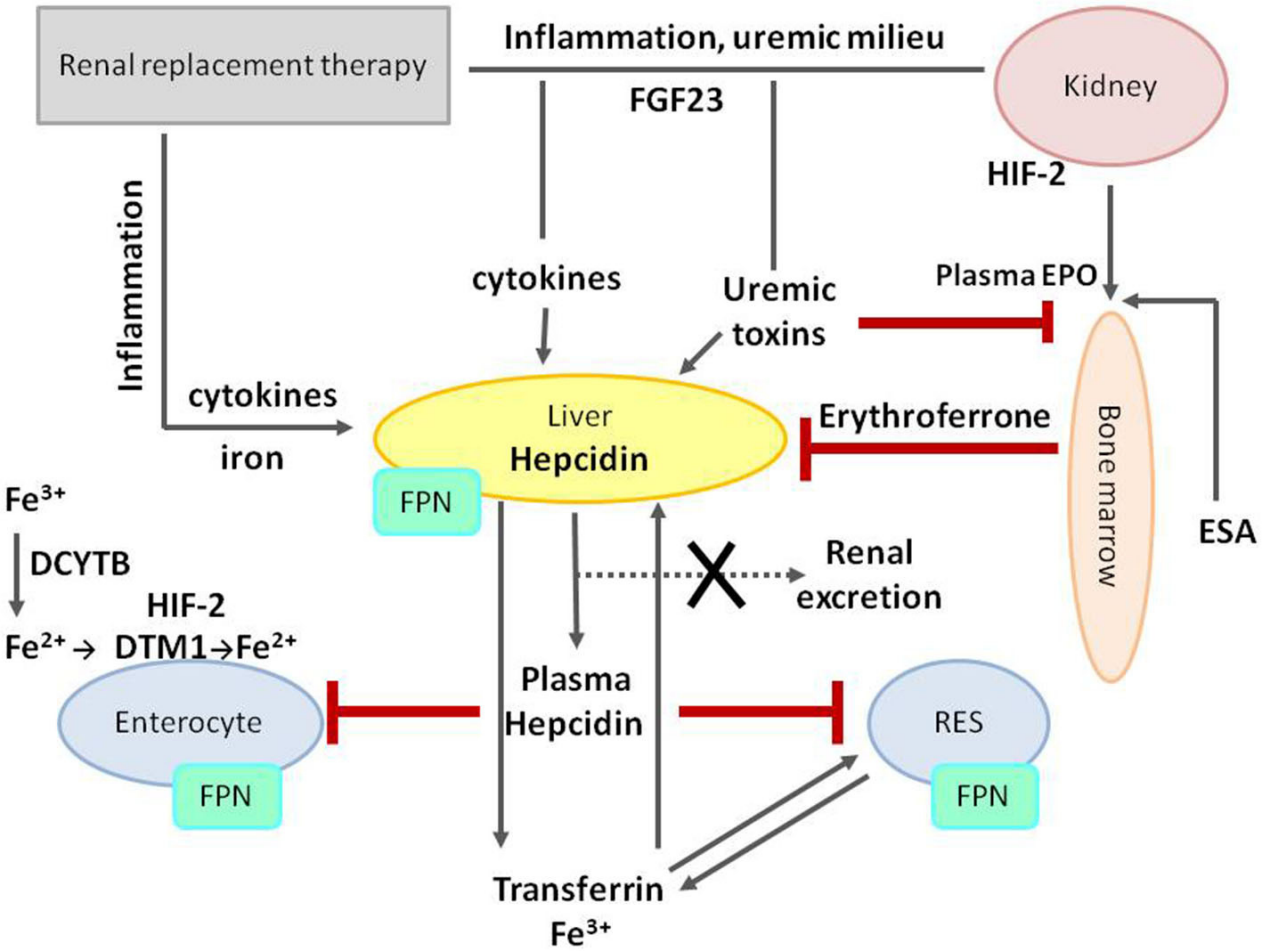
Iron metabolism dysregulation in chronic kidney disease. The central role in iron metabolism regulation plays an increased level of hepcidin, decreasing FPN on cell membranes and thus diminishing duodenal iron absorption and acquirement from stores in RES. Hepcidin expression and production are stimulated by iron treatment, inflammation, uremic milieu, and fibroblast growth factor 23 (FGF23), and, at the same time, its removal is impaired due to decreased glomerular filtration rate (GFR). Both erythropoietin and erythroferrone play suppressive effect on hepcidin production. FPN, ferroportin; RES, reticuloendothelial system; HIF-2, hypoxia-inducible factor 2; DCYTB, duodenal cytochrome B reductase; DTM1, divalent metal transporter 1; FGF23, fibroblast growth factor 23; EPO, erythropoietin; ESA, erythropoiesis-stimulating agent.

The hepcidin–ferroportin axis has a central role in iron balance regulation and cooperation with hypoxia responsive pathways and EPO signaling. Many factors related to CKD may affect the functioning of the hepcidin–ferroportin axis, as well as each stage of iron trafficking, as shown in Figure 6.

### Erythropoiesis in Chronic Kidney Disease

In CKD, the mechanism of regulation of EPO secretion in response to the hypoxic stimulus remains fairly long functional, and EPO levels may be normal or even slightly increased, however inappropriately low relative to the degree of anemia (2, 3). Along with the loss of renal parenchyma, the efficiency of EPO production gradually decreases, and its deficiency becomes more pronounced. Moreover, increasing levels of kynurenine (product of l-tryptophan) and indoxyl sulfate (uremic toxin) are observed in uremia, aryl hydrocarbon receptor nucleus translocator (ARNT) is activated and competes with HIF-2α to prevent its binding to HIF-β, and EPO production is decreasing (24, 25). In addition, excess iron reduces HIF-2α and EPO expression in the event of EPO deficiency (26). These factors simultaneously stimulate expression and production of hepcidin, a critical factor in iron dysregulation in CKD.

The fact that anemia in CKD develops even despite of elevated EPO levels may also result from peripheral EPO hyporesponsiveness or resistance caused by inflammation or secondary hyperparathyroidism (2, 3).

### Erythroferrone—The Link Between Erythropoiesis and Iron Metabolism in Chronic Kidney Disease

In CKD patients who have relative increase of endogenous serum EPO levels or are treated with exogenous EPO, ERFE concentrations are increased. ERFE can potentially interact with the hepcidin rising effects of inflammation and impaired hepcidin clearance and may play a role in the modulation of iron homeostasis in CKD patients. However, ERFE concentrations and actions are not well-characterized in CKD patients. As yet, information on ERFE in CKD is scarce and, in fact, limited to three studies, which yield slightly inconsistent results (27–29). All of them confirmed positive correlations between serum ERFE levels and serum EPO, as well as ESA dose, while the relationships between ERFE and biomarkers of iron metabolism have not been clearly demonstrated (27–29). In the Honda et al. study of hemodialysis (HD) patients treated with long-acting ESAs (darbepoetin-α or continuous EPO receptor activator), levels of ERFE increased shortly after ESA injection, and hepcidin levels decreased few hours after ESA stimulation. A statistically significant negative correlation between ERFE and hepcidin and ferritin levels, and a positive correlation with soluble transferrin receptor were found. It suggests that ERFE may be a key regulator of iron mobilization from body stores during erythropoiesis stimulated by ESA and could be useful for monitoring iron metabolism in CKD patients (27). The inverse relationship between ERFE and biomarkers of iron metabolism (serum iron and ferritin) was confirmed in the cohort of CKD and HD patients in the Spoto et al. study (29). Meanwhile, in the Honda et al. study, ERFE correlated positively with serum EPO level in non-dialysis CKD patients not treated with ESA, while in dialysis-dependent CKD patients, the positive correlation with ESA dose was found. However, in neither cohort of this study did ERFE correlate with hepcidin or any biomarker of iron metabolism. This could suggest that pathological modulation of hepcidin in CKD by inflammation, uremic milieu, or impaired clearance may mask or decrease the hepcidin inhibitory effect of ERFE (28). The modulatory effect of ERFE on iron homeostasis, as well as the utility of ERFE as a biomarker of erythropoietic activity in CKD population, must be explained in future studies.

Interestingly, in the Spoto et al. study, serum ERFE was associated with clinical outcomes (all-cause mortality and non-fatal CV events) in predialysis CKD and HD patients, independently of confounders like C-reactive protein (CRP) or iron metabolism biomarkers (29). This could suggest that EPO by increasing the ERFE concentration may amplify the risk of cardiovascular complications in CKD patients, but this hypothesized relationship has to be demonstrated and confirmed in large-scale studies.

### Hepcidin–Ferroportin Axis—A Key Regulator of Iron Balance in Chronic Kidney Disease

The functioning of the hepcidin–ferroportin axis is disturbed in patients with CKD, resulting in a disarrangement in the coordination of acquisition, storage, and iron utilization at the cellular and systemic levels. In general, downregulation of ferroportin and excess hepcidin promote iron restriction and limit its absorption, availability for recycling, and delivery to erythroid precursors for hemoglobin synthesis (12). Ferroportin mediates all major iron flows, and the loss of ferroportin from cell surfaces decreases the delivery of iron from cells to blood plasma.

Due to this export block, absorbed dietary iron cannot be transferred from duodenal enterocytes to plasma, is retained in the cells, and then lost during shedding. In addition to this mechanism, in CKD patients, iron absorption may be impaired due to insufficient dietary supply and the use of phosphate binders typically used to treat hyperphosphatemia.

The same mechanism is involved in iron restriction within the reticuloendothelial system. The loss of ferroportin blocks the release of recycled iron from macrophages, enhances retention, and hinders mobilization from stores in the presence of increased demands.

Parenteral iron administration, often used in patients with CKD, stimulates hepcidin expression and may paradoxically worsen iron restriction.

### Hepcidin in Chronic Kidney Disease

In CKD patients, inflammatory state originating from excessive generation and retention of uremic toxins, exposure to catheters, dialysis membranes, and fluids increases production of hepcidin. This increase in circulating hepcidin level is crucial to disordered iron availability and supply for erythropoiesis.

In CKD patients, hepcidin levels are significantly increased, even up to 9-fold in HD patients (30). Impaired renal clearance, scarce elimination by dialysis, chronic inflammation, and, paradoxically, parenteral iron administration may be involved in the increase.

Serum hepcidin levels inversely correlate with eGFR; however, this association is evident only in advanced (G3b-5) CKD stages (31). This suggests that reduced clearance may contribute to elevated serum hepcidin levels only in advanced but not early stages of CKD and that the pathogenesis of elevated serum hepcidin levels may be different in these patients (31). Nevertheless, renal clearance may be a relevant way of hepcidin elimination in end-stage renal disease patients since individuals with preserved residual renal function have lower serum hepcidin levels irrespective of the dialysis method (32). The influence of other factors cannot be ruled out because anuric patients have more pronounced inflammation and oxidative stress, both stimulating hepcidin production.

In dialyzed patients, hepcidin as a middle-molecular-weight substance (molecular weight 2.71 kDa) may be eliminated by dialysis; however, its serum concentration rebounds in few hours after HD procedure cessation (30). The clearance of hepcidin depends on the following: (1) the permeability of the membrane used, increasing from weak in low-flux HD to middle in high-flux HD and (2) the type of transport, increased in online hemodiafiltration (OL-HDF) due to dominating convective transport (33). It was demonstrated that the use of more efficient techniques allows for not only the removal of substantial amount of hepcidin but also a significant reduction in pre-HD hepcidin levels. Using high volume OL-HDF, Stefansson et al. obtained a significant (38%) reduction of serum hepcidin levels after 60 days of treatment (34). In turn, in the Teatini et al. study, the use of high cutoff membrane confirmed the removal of a high quantity of hepcidin and a 50% reduction its pre-dialysis serum level (35). The application of convective transport and good membrane permeability appears to be the solution to obtain lower levels of hepcidin in dialysis patients; however, it should be confirmed in future large-scale studies.

Peritoneal dialysis (PD) is also effective in hepcidin elimination; however, data regarding the efficacy are scarce. In only one study, the effect of PD on serum hepcidin level in HD compared with HDF was studied (32). It was demonstrated that PD patients have significantly lower serum hepcidin levels than HD patients compared with HDF patients. Moreover, PD seems to provide effective removal of hepcidin via peritoneal membrane because concentration of hepcidin in peritoneal effluent was higher than that of HD and HDF ultrafiltrate (32). This observation appears to be due to the physiology of PD. PD is a continuous technique with diffusive and convective transport of small- and middle-molecular solutes; however, individual properties of peritoneal membrane may influence efficacy of hepcidin removal. So far, peritoneal hepcidin clearance using peritoneal equilibration test was evaluated only in one small study (36). The efficacy of PD removal should be confirmed in further studies. Also, it seems necessary to examine how hepcidin is removed through the peritoneal membrane and whether the type of peritoneal transport has an effect on it.

Iron administration, apart from ESAs, remains the cornerstone of anemia management in CKD patients. However, considering iron metabolism's pathophysiology in CKD, iron *per se* stimulates hepcidin expression and ferroportin downregulation, paradoxically worsens iron restriction, potentiates functional iron deficiency, and reduces ESA responsiveness (11).

Recent research has revealed bidirectional relationships between bone-secreted fibroblast growth factor 23 (FGF23), inflammation, iron status, and anemia. Classically described regulators of FGF23 include phosphate, calcium, vitamin D, and parathyroid hormone; however, new actions of FGF23 beyond its mineral homeostasis regulation have been described; and inflammation, iron status, and EPO have emerged as new players participating in FGF23 regulation. Iron deficiency, inflammation, and EPO have been shown to increase FGF23 levels and cleavage; and conversely, FGF23, particularly in CKD, may promote anemia, iron deficiency, and inflammation (37–39). In one of the recent studies, log-transformed total FGF23 was inversely associated with serum iron and TSAT and positively with hepcidin; and associations between FGF23 and anemia persisted after adjustment for ferritin and CRP (40). In another study, including HD patients, EPO dose was positively associated with log total FGF23, even after adjustment for TSAT, ferritin, hemoglobin, and CRP (41).

## From Understanding Pathophysiology of Iron Metabolism to Novel Therapies

In accordance with growing evidence from randomized clinical trials and observational studies, iron administration remains a cornerstone of anemia management in CKD patients. Considering the limited effectiveness of oral supplementation, especially in dialysis-dependent CKD patients, the preferred form is intravenous administration. Despite proven efficacy in iron repletion, ESA sparing, and correcting anemia, many concerns remain regarding parenteral iron's long-term safety, especially when administering high or repeated doses, as recently reviewed (42, 43). It is still unclear whether oxidative stress induced by intravenous iron infusion may lead to an increased risk of infection, atherosclerosis, cardiovascular complications, and mortality, as well as tissue iron overload and organ damage. Several studies evaluating this topic yield inconsistent or even opposite results (42, 43). Furthermore, considering the pathophysiology of iron metabolism in CKD, iron, which *per se* stimulates hepcidin expression and ferroportin downregulation, may paradoxically worsen iron restriction, potentiate functional iron deficiency, and reduce ESA responsiveness (11, 13).

An interesting and promising method in IDA management in CKD patients appears to be novel oral iron preparations (44). One of these agents, ferric citrate, initially evaluated as phosphate binder, was demonstrated to improve iron parameters and anemia correction in dialysis-dependent and predialysis CKD patients (45–47). Moreover, ferric citrate showed substantial reduction in ESAs and parenteral iron use in dialysis-dependent CKD patients (48). Additional trials, however, are needed to assess the long-term effects of ferric citrate as an iron-repletion agent. Other novel oral iron preparations, ferric maltol and sucrosomial iron, are in development to treat IDA in CKD patients (44).

Moreover, increasing knowledge on iron physiology and pathophysiology allows for the identification of numerous novel therapeutic targets. The understanding that hepcidin is a key master in developing iron-restricted erythropoiesis caused interest in the new therapies that target more directly dysfunction of hepcidin–ferroportin axis.

Some of these agents already entered human clinical trials, and some are under development, as they have shown efficacy in animal models of chronic disease.

### Hepcidin Synthesis Inhibitors

Two signaling pathways have been fixed on inhibition of hepcidin synthesis targeting the positive regulators: BMP6–HJV–SMAD pathway inhibitors and IL-6–STAT3 pathway inhibitors. Dorsomorphin, a small molecule inhibitor of BMP receptor type I kinases, was shown to inhibit BMP–HJV- and IL-6-stimulated hepcidin expression in cultured hepatocytes and to block iron-induced hepcidin mRNA in mice. However, dorsomorphin is a non-selective kinase inhibitor and inhibits AMP kinase. A derivative of dorsomorphin, LDN-193189, was shown to have improved potency and selectivity as a BMP inhibitor. Over 4 weeks, it reduced hepatic hepcidin mRNA levels, increased serum iron concentration, increased ferroportin expression in splenic macrophages, and, importantly, improved hemoglobin levels and hematocrit in anemic rats. However, LDN-193189 potently inhibits vascular endothelial growth factor (VEGF) and components of the mitogen-activated protein kinase–extracellular signal-regulated kinase (MAPK–ERK) pathway. The results from studies using LDN-193189 must be interpreted with caution (49, 50).

Inhibitors of the IL-6 pathway have been shown to downregulate hepcidin expression and improve anemia of inflammation in multicentric Castleman's disease. Treatment with the anti-IL-6 receptor antibody (anti-IL-6R) tocilizumab for 6–12 months decreased serum hepcidin levels and normalized blood hemoglobin (51).

Blocking antibodies to the IL-6 ligand also resulted in similar hepcidin-lowering effects. The anti-IL-6 chimeric monoclonal antibody, siltuximab, was recently demonstrated to increase hemoglobin levels by 2.1 g/dl, but it has been associated with increased risk of infections (52).

### Hepcidin–Ferroportin Axis (Ferroportin Agonists/Stabilizers)

This axis can be targeted at various points. Hepcidin antibodies have been shown to bind human (and monkey) hepcidin, inhibit its action on ferroportin, enhance the dietary absorption, and promote mobilization of iron from stores (53).

The ferroportin stabilizers tend to reduce hepcidin expression, inhibit its action, and prevent ferroportin degradation. Anti-ferroportin monoclonal antibody (LY2928057) blocks hepcidin's interaction with its receptor, thereby reducing ferroportin internalization and allowing more iron flux. In CKD patients, the administration of LY2928057 resulted in an increase in hemoglobin and reduction in ferritin (53).

### Targeting Hypoxia-Inducible Factor Inhibitors

HIF's stabilization via a propyl hydoxylase inhibitor (HIFα-PHI) may be an effective therapeutic target in the treatment of anemia of CKD. HIF-PHIs induce activation of the genes responsible for erythropoiesis, creating “pseudohypoxic” state and allow for HIF-α/β dimerization and signal transduction through the HIF pathway resulting in effects on EPO andiron balance (54). The main results of studies with HIF-PHIs are summarized in Table 1.

**Table 1:** The main results of selected trials with HIF-PHIs in CKD patients.

**Substance**	**Study design and population**	**Most important findings**	**References**
Roxadustat	NDD-CKD patients; various dose regimens	In 92% of patients, Hb response independent of baseline CRP and iron repletion 16.9% reduction in serum hepcidin level	(55)
	ESA-naive incident PD and HD patients; no iron or oral iron or iv iron + roxadustat	In 96% of patients, Hb response independent of baseline Hb, iron-repletion status, inflammatory status, and dialysis modality >40% reduction in serum hepcidin level	(56)
Molidustat	ND-CKD patients; switch from darbepoetin to molidustat or continued with darbepoetin	Maintaining Hb level within the target range after switching from ESA	(57)
	D-CKD patients; switch from epoetin to molidustat or continued with epoetin	Maintaining Hb level within the target range after switching from ESA	(57)
Vadadustat	NDD-CKD patients; escalating vadadustat doses vs. placebo	Increasing Hb in dose-dependent manner Increasing TIBC, decreasing ferritin, and hepcidin levels	(58)
Daprodustat	CKD (stage 3–5) patients; fixed daprodustat doses vs. placebo HD patients; daprodustat vs. rhEPO continuation	Dose-dependent increase in Hb and dose-dependent decrease in hepcidin level 5 mg of daprodustat allowed maintaining Hb levels similarly to rhEPO; when given in lower doses, Hb levels decreased	(59)

*HIF, hypoxia-inducible factor; PHI, propyl hydoxylase inhibitor; CKD, chronic kidney disease; NDD, non-dialysis dependent; CRP, C-reactive protein; ESA, erythropoiesis-stimulating agent; TIBC, total iron-binding capacity*.

The treatment with HIF-PHIs raises some safety concerns. In short-term studies, no serious adverse events were reported. However, longer follow-up periods are warranted to validate the safety of prolonged HIF elevation, considering its role in fibrogenesis and inflammation responses, as well as possible risk in tumorigenesis or vascular calcification. Studies with longer follow-up and maybe a weighed dosing strategy are needed to ensure the safety of the drugs in long-term use (54, 60).

### Other Compounds

Anticalins are proteins with engineered ligand-binding properties derived from the human's lipocalin. Lipocalins are low-molecular-weight widespread proteins that transport, store, or sequester small biological compounds like vitamins or hormones. Anticalin PRS-080#22 has been shown to sequester hepcidin. In CKD patients on HD, anticalin PRS-080#22 decreased serum hepcidin and increased serum iron and TSAT in a dose-dependent manner (61).

The inhibition of the FGF23 signaling pathway increased erythropoiesis, serum iron, and ferritin levels and reduced inflammation and erythroid apoptosis (62).

## Conclusion

Anemia is one of the most typical features of CKD for over 150 years. In the past, blood transfusions and iron preparations were the only therapeutic options, whereas nowadays, ESAs and iron are mainstay in the treatment of renal anemia. Understanding the mechanisms underlying dysregulation of iron balance in CKD patients is holding the promise for development of new therapeutic strategies to improve anemia management, involving hepcidin and hepcidin–ferroportin axis as well as prolyl hydroxylase inhibitors and modulators of hepcidin activity. It enhances the possibility of more precise control of current iron therapies as well as new pharmacological and non-pharmacological hepcidin-reducing approaches, including various methods of renal replacement therapy. As iron is supposed to have Janus's face, iron status modulation is vital in kidney patients. Nevertheless, efficacy and long-term safety of new strategies should be confirmed in prospective controlled trials, as populations of patients with CKD are getting older with more comorbidities or even multimorbidities.

## Author Contributions

JM and EW conceived the idea for the study and contributed to the design of the research. JM, EW, and TG were involved in the preparation of the manuscript. All the authors analyzed the data and edited and approved the final version of the manuscript.

## Conflict of Interest

The authors declare that the research was conducted in the absence of any commercial or financial relationships that could be construed as a potential conflict of interest.
